# Visualising Silicon in Plants: Histochemistry, Silica Sculptures and Elemental Imaging

**DOI:** 10.3390/cells9041066

**Published:** 2020-04-24

**Authors:** Gea Guerriero, Ian Stokes, Nathalie Valle, Jean-Francois Hausman, Christopher Exley

**Affiliations:** 1 Environmental Research and Innovation (ERIN) Department, Luxembourg Institute of Science and Technology, 5, rue Bommel, Z.A.E. Robert Steichen, L-4940 Hautcharage, Luxembourg; jean-francois.hausman@list.lu; 2 The Birchall Centre, Lennard-Jones Laboratories, Keele University, Keele ST5 5BG, Staffordshire, UK; ian.d.stokes@gmail.com; 3 Material Research and Technology (MRT) Department, Luxembourg Institute of Science and Technology, 41 rue du Brill, L-4422 Belvaux, Luxembourg; nathalie.valle@list.lu

**Keywords:** biogenic silica, microscopy, PDMPO, SEM, microwave acid digestion, elemental imaging

## Abstract

Silicon is a non-essential element for plants and is available in biota as silicic acid. Its presence has been associated with a general improvement of plant vigour and response to exogenous stresses. Plants accumulate silicon in their tissues as amorphous silica and cell walls are preferential sites. While several papers have been published on the mitigatory effects that silicon has on plants under stress, there has been less research on imaging silicon in plant tissues. Imaging offers important complementary results to molecular data, since it provides spatial information. Herein, the focus is on histochemistry coupled to optical microscopy, fluorescence and scanning electron microscopy of microwave acid extracted plant silica, techniques based on particle-induced X-ray emission, X-ray fluorescence spectrometry and mass spectrometry imaging (NanoSIMS). Sample preparation procedures will not be discussed in detail, as several reviews have already treated this subject extensively. We focus instead on the information that each technique provides by offering, for each imaging approach, examples from both silicifiers (giant horsetail and rice) and non-accumulators (*Cannabis sativa* L.).

## 1. Introduction

Silicon (Si) is the second most abundant element on Earth after oxygen and it is considered non-essential for plant growth and development [1]. Si is beneficial and, when provided to plants, it increases vigour, resistance to environmental stresses and mechanical strength of aerial organs [2,3]. The form of Si that is taken up by plants is silicic acid-Si(OH)_4_, a very weak acid with pKa of ca. 9.5. The entry route from the soil follows water [4] and additionally uses channels of the aquaporin family (Nod26-like intrinsic proteins, NIPs) [5]. Members of this family of channels have been discovered in several plants [6,7], among them the fibre crops flax and hemp [8,9].

With respect to Si accumulation, plants are distinguished into excluders (Si <0.5% in dry weight), accumulators (Si > 1% in dry weight) and intermediate types (Si between 0.5 and 1% in dry weight) [10,11,12]: among the accumulators are Equisetales, Cyperales and Poales, while tomato and other members of the nightshade family are Si-excluders [13]. Si associates with plant tissues as amorphous hydrated opaline silica (SiO_2_ × nH_2_O) and the process of biogenic silica precipitation is referred to as silicification. 

It is known that silica associates with the cell wall and that specific macromolecules can template its deposition [2]. Cellulosic, non-cellulosic polysaccharides and the aromatic macromolecule lignin were shown to be associated with silica deposition [14,15,16,17]. A clear proof of the role of another cell wall polysaccharide, i.e., callose, in templating silica deposition is found in the study by Law and Exley [18], where the authors showed, both *in vitro* (using an undersaturated solution of silicic acid) and *in vivo*, that silicification in *Equisetum arvense* follows callose deposition. Indeed, horsetail silica is found intimately associated with callose in different cell types [19]. The role of callose in silicification has been further confirmed in other species, namely rice [20] and *Arabidopsis thaliana* [21], where plants lacking the callose synthase gene *POWDERY MILDEW RESISTANT 4*-*PMR4* accumulated significantly less silica, as compared to wild-type and overexpressing plants [22]. The cell wall templating mechanism may be passive and supported by the gel-like nature of specific cell wall components (like callose), which trap silicic acid in a microenvironment favourable for auto-condensation into silica [18]. Yet, it is not known whether silicification ‘prefers’ specific cell wall macromolecules or if the process can be triggered by any type of cell wall macromolecule. 

The study of metal(loid) uptake and accumulation in plants has received much interest in the last years [23,24,25,26], as they affect important physiological processes in plants, impact yield and, in the case of crops used for food and feed, can raise health-related issues. The study of metal(loid) accumulation in plants needs imaging to gain information on the sites of accumulation, on distribution and translocation routes. The development of tools coupling spatial resolution to the analysis of speciation is needed for a complete understanding of their metabolism [27]. In addition, techniques enabling *in vivo* detection in plants add information in real-time, which eliminate the bias caused by sample preparation.

In this review, imaging techniques to study plant silicification are discussed with an emphasis on their advantages and drawbacks. Classical histochemistry with known stains/fluorophores is discussed together with techniques implying the extraction of plant silica and subsequent scanning electron microscopy (SEM), X-ray emission spectrometry (micro-PIXE microbeam particle-induced X-ray emission, LEXRF low-energy X-ray fluorescence) and mass spectrometry (SIMS-secondary ion mass spectrometry nano-analysis). Although other analytical techniques like Raman microscopy or micro computed tomography (MicroCT) have already shown their suitability to study the distribution of Si in plants, we have confined our investigations to histochemistry, SEM, X-ray emission spectroscopy and mass spectrometry imaging. More details about the use of Raman imaging applied to plant research can be found in [28]. MicroCT is more effective for the analysis of large phytoliths [29] and used in archaeobotanical studies [30], while Raman is useful to combine spatial information with chemical composition.

A summary of the techniques surveyed is provided in Table 1. Examples of their utility in studying plant silicification are provided by case studies on both heavy silicifiers (such as rice and horsetail) and low silica accumulators (textile hemp).

## 2. Dyes and Fluorophores to Visualize Plant Silica

Plant silica can be observed with light microscopy after staining with methyl red (MR), crystal violet lactone (CVL) and silver-amine chromate (SAC) [36]. Amorphous silica adsorbs MR and CVL from non-polar solvents which then confer red and blue/violet colour, respectively. The azo dye MR is normally used to measure surface polarity and provides an indication of residual silanol groups [37]. The dye (usually the acid form which stains more intensively than the sodium salt or the hydrochloride [36]) dissolved in dilute benzene is orange in colour and turns red upon adsorption to acidic groups, such as silanols. Although fast green (staining in green cellulose and the cytoplasm) can be used as counterstain for MR, both lignin and silica will be red when visualised under the light microscope [38]. This is a drawback when studying lignified and silicified tissues, as there is no possibility to distinguish them.

CVL (usually 0.1% w/v in benzene [36,39]) stains silica via the formation of hydrogen bonds between the carboxylic group of the dye and the silanols. This staining is used to visualize the silicified cells of rice [39] and to study Si uptake in mutants [40]. Safranin can be used as a counterstain, but as discussed for MR, there is a lack of specificity, since lignin is also stained violet [38]. 

MR and CVL give variability in staining; indeed, for specific cell types, like bulliform and silica cells, staining will not be visible, unless etching is done (the process of treating the samples with an acid, hydrofluoric acid-HF for example, to free the silicified structures from the organic material [36]). 

It was suggested that the outer layer of these cells may be packed with silica and have tight surface pore sizes hindering the penetration of the dye [36]. The reactivity of these dyes depends on the accessible silanol groups; not all silanol groups within silica may be accessible due to steric hindrance or may be buried within the silica layer. 

SAC (prepared by mixing AgNO_3_ with K_2_CrO_4_, then washing the precipitate in hot water at 50–60 °C and dissolving it in 3% v/v NH_4_OH, followed by filtering) gives a brown colour upon adsorption to amorphous silica due to the removal of ammonia and the unmasking of silver chromate [36]. This staining was used to observe silicified cells, such as bulliform cells of rice [36] and venuloid idioblasts from *Pteris* [41]. Treatment in H_2_SO_4_ prior to SAC incubation enhances the intensity of the staining because of the partial digestion of neighbouring cells which facilitates penetration of the solution; however, excess acids may cause unspecific precipitation of SAC [36]. After SAC staining, washing and dehydration in an alcohol series can be performed to increase the contrast of the stained cells which turn from red-brown to black [36]. Histochemical staining of plant silica, despite being relatively easy to perform and relying on instruments that are accessible in any laboratory, requires invasive treatments, namely tissue fixation and, eventually, etching. The resolution is quite low and not selective enough to discriminate for example lignified tissues. 

Being able to distinguish silicified and lignified tissues is essential to understand the role(s) of lignin and silica in different plant species and/or under different growth conditions. For example, silica and lignin are two compounds expected to play a mechanical role of support in plant tissues. Silica is however a cheaper alternative to lignin [42,43], requiring a lower energetic investment as compared to the C-based macromolecule lignin; therefore, plants relying on silica for structural support should theoretically be able to shunt more energy toward primary production than plants synthesizing lignin. Understanding the relationship between silica/lignin accumulation and primary productivity in plants is an important topic in ecology; hence, it is necessary to distinguish between lignified and silicified tissues in plant specimens. 

Biosilicification can be visualised using a fluorescence microscope and the fluorophore 2-(4-pyridyl)-5-((4-(2-dimethylaminoethylaminocarbamoyl)methoxy)phenyl)oxazole (PDMPO). PDMPO is a silica tracer used to monitor pH changes within acidic organelles [44]. The fluorophore has been extensively used to monitor silicification in diatoms [45], as well as to analyse the fine details of extracted plant silica [9,18,19,20,22] and analyse it *in planta* [29].

**Figure 1:**
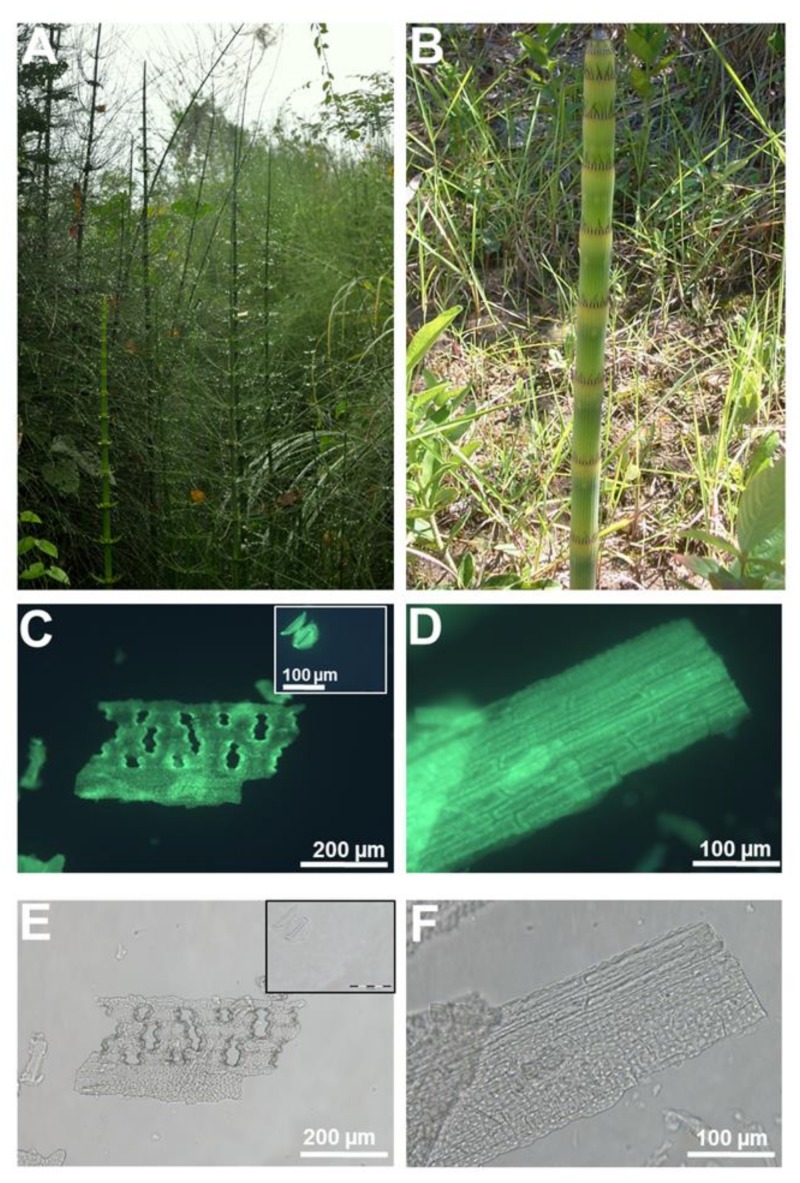
Heavily silicified giant horsetail (*Equisetum myriochaetum* L.). (**A**,**B**) Giant horsetail growing in the wild (Peruvian Amazon). (**C**) Biogenic silica in giant horsetail’s stem following acid extraction and fluorescence imaging with 2-(4-pyridyl)-5-((4-(2-dimethylaminoethylaminocarbamoyl)methoxy)phenyl)oxazole (PDMPO). Holes corresponding to sunken stomata are visible. Inset: heavily silicified stomata with fine details of radiating ribs in the guard cells. (**D**) Undulating silicified cell walls of the epidermal cells on the stem. (**E**,**F**) Differential interference contrast images of (**C**) and (**D**). Images were taken using an Olympus BX50 fluorescence microscope (Southend-on-Sea, UK).

**Figure 2:**
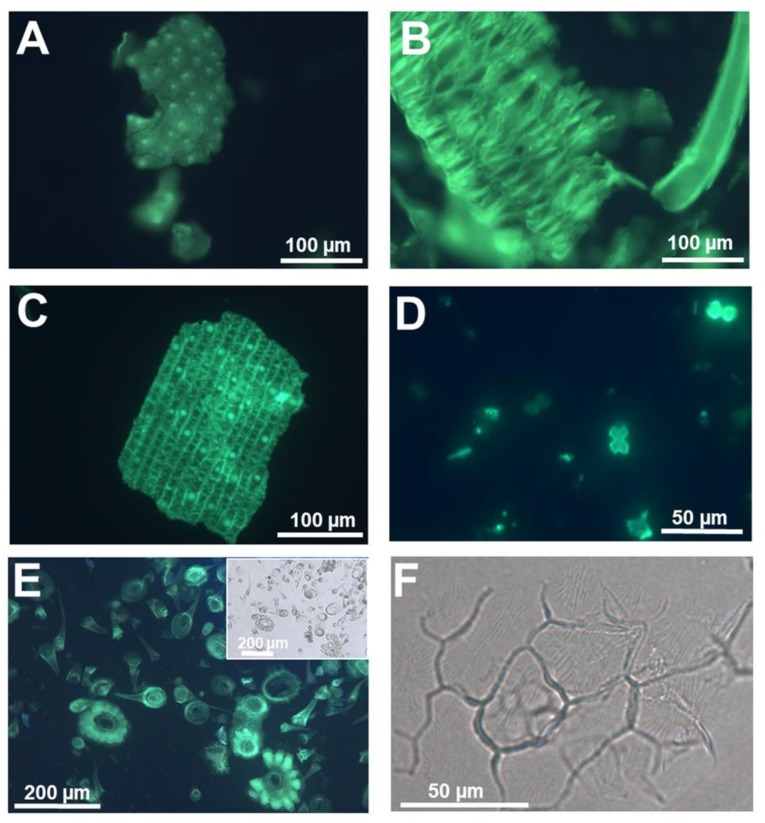
Silica extracted from a silicifier (*Oryza sativa* L.) and non-silicifier (*Cannabis sativa* L.) (**A**) Silica stained with PDMPO and extracted from rice seedlings at the 4^th^ leaf stage. Punctate silicified warts are present on the surface. (**B**) Silica stained with PDMPO and extracted from rice seedlings at the 3^rd^ leaf stage. The feathery elements are likely whiskers on the surface of husks. (**C**) Silica from rice leaf epidermis showing silicified papillae, nodules and interlocking sinuate pattern of the epidermal cell walls. (**D**) Silica and bulliform cells from rice leaf epidermis. (**E**) PDMPO staining of silica collected following acid and microwave digestion of hemp leaves and showing trichomes with/without basal cells. Inset: differential interference contrast image. (**F**) Differential interference contrast image of silica extracted from hemp leaves and showing a group of epidermal cells with details of the waxy wrinkles on the surface. Images were taken using an Olympus BX50 fluorescence microscope.

For example, we have used PDMPO staining of silica extracted after removal of organic material to study the fine cellular details of the silicified cell replicas in both Si accumulators and non-accumulators (Figure 1 and Figure 2).

Microwave acid digestion proved to be a suitable method to free inorganic silica deposits from organic plant material [9,18,19,20,22]. PDMPO staining of live tissues can provide complementary information to the one obtained from extracted silica. The specificity of the signal in different cell types with, e.g., different degrees of lignification needs to be checked. Pierantoni and colleagues (2017) showed the suitability of using PDMPO in okra leaves and revealed the presence of small particles which are normally lost during phytoliths’ extraction [29].

During acid digestion, the plant sample is exposed to a cocktail of highly corrosive acids (HNO_3_ and H_2_SO_4_) within sealed containers. These containers are then subjected to a microwave digestion program (180 °C for 15 minutes and maintained at 180 °C for 30 minutes) which breaks down the sample matrix leaving behind its component elements in solution. It was demonstrated that, although this procedure successfully digests all organic plant tissue, the inorganic silica structures are left intact [18]. Silica can thereafter be collected after filtering through a 0.2 μm filter (this step may cause loss of smaller particles, such as those described in Pierantoni et al. [29]) and several washes in ultrapure water and either be stained with PDMPO or observed with SEM (see next section).

The silica extracted from giant horsetail (Figure 1A,B) shows extremely fine details of papillae, pores and stomata (both sunken and not, Figure 1C–E and insets), as well as the undulating cell walls of the epidermal cells of the stem (Figure 1D,F). 

Likewise, in rice, details of the silicified warts (Figure 2A), whiskers on the surface of the husks (Figure 2B) and the sinuate pattern of the walls of epidermal cells showing a mosaic-like structure can also be clearly observed (Figure 2C). Isolated silica and bulliform cells (Figure 2D) are abundant in silica extracted from rice tissues. 

PDMPO staining of extracted silica was also recently used in non-accumulators. Textile hemp does not accumulate the same levels of silica as horsetail or rice; nevertheless, when grown in the presence of sodium metasilicate, silicified trichomes and leaf epidermal cells are visible [9] (Figure 2E,F). The same richness in details described above for horsetail and rice is present in hemp silica: particularly detailed are the trichome structures, where the wrinkles on the waxy surface of basal cells are perfectly replicated as silica (Figure 2F; [9]).

PDMPO staining was also recently applied to the observation of fresh okra leaves by confocal microscopy [29]. The fluorophore was infiltrated in the leaves by immersing sections in the solution containing PDMPO and by applying a vacuum for 30 minutes. It would be useful to study silicification in the leaves of mutants impaired in the synthesis of cell wall components, such as callose, cellulose, non-hemicellulosic polysaccharides, or pectin. In this respect, the wide collection of thale cress mutants is an important resource and would allow a rapid screening after PDMPO infiltration. *Arabidopsis* was indeed shown to accumulate Si when grown in the presence of the metalloid [22] and a comparative study of the different cell wall mutants available would provide insights into which cell wall component is capable of templating silica deposition.

## 3. Micromorphology: Scanning Electron Microscopy of Extracted Plant Silica

High-resolution microscopy, such as SEM, can be used to image plant silica and estimate the extent of silicification by observing tissue and cell types at the nanometer scale. From the observation of silicified cell types, it is possible to infer the functional role of silica. The high resolution of SEM allows analysis of micromorphology at the cell level and of the surface microstructure in silicified tissues. Silicified ‘microsculptures’ from horsetail were already imaged with SEM almost three decades ago [46]: it was possible to show the intimate relationship of biosilicification with the secondary cell walls and the incredible heterogeneity of silicified structures. The removal of organic material present, e.g., on the epidermal surface unmasks the fine details of the surface texture and provides a higher definition [46].

**Figure 3:**
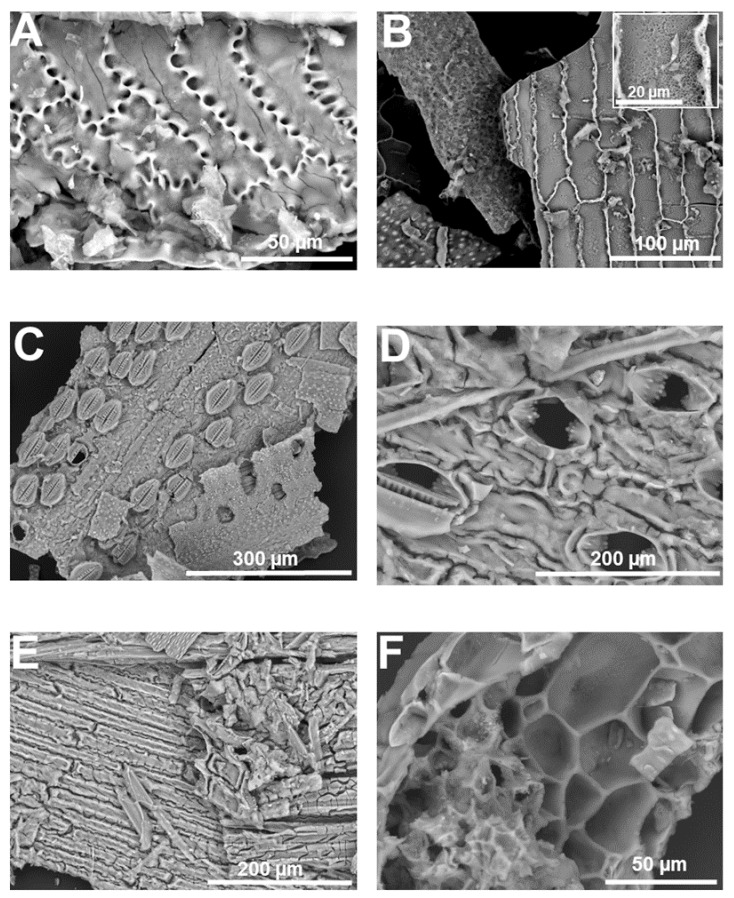
SEM images (topographic contrast) of silica extracted from giant horsetail’s stem. (**A**) Jigsaw-puzzle-like epidermal cells with interdigitated lobes. (**B**) Epidermal cells with undulating walls showing a porous siliceous structure. Inset: detail of the porous texture of the extracted silica. (**C**) Stomata with very fine cellular details of the radiating ribs and sunken stomata among silicified papillae on the stem surface. (**D**) Fine siliceous replica of stomata pores, with details of silicified projections of the outer ledge. (**E**) Epidermal cells and fibrous material extracted from giant horsetail’s stem. (**F**) Probable parenchyma cells. Images were taken using a HITACHI TM3000 TableTop scanning electron microscope.

By using microwave acid digestion followed by SEM observations (both a benchtop SEM designed for ease of use and minimal sample preparation and a conventional one providing higher resolution), a staggering heterogeneity of silicified cell types can be observed in giant horsetail (Figure 3).

**Figure 4:**
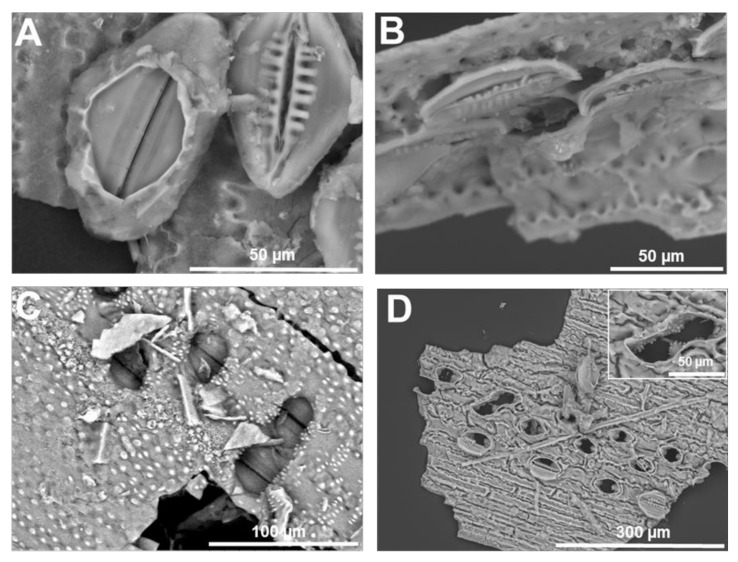
SEM images (topographic contrast) of different typologies of stomata on the stem of giant horsetail. (**A**) Two stomata possibly at different developmental stages (the one on the left is covered by a layer and is devoid of radiating ribs, while the stomata on the right is mature). (**B**) Transversal view of an adult stomata cut in half with details of the radiating ribs and neighbouring epidermal cells. (**C**) Sunken stomata interspersed among silicified papillae. (**D**) Naked and/or partially covered stomatal pores. Inset: detail of a bigger distorted stomatal pore with silicified projections. Images were taken using a HITACHI TM3000 TableTop scanning electron microscope.

The interdigitated lobes of the epidermal cells of the stem are perfectly replicated, together with recesses running all along the undulating margins (Figure 3A); in some extracted samples, it is possible to observe the microstructure reminiscent of a porous rock (Figure 3B). The stomata, sunken or not, covering the stem are replicated in even the smallest structural detail (Figure 3C): the radiating ribs are clearly visible and, in some extracted silica, the super-adjacent guard and subsidiary cells [47] unmask the underlying pore opening, thereby revealing the silicified projections protruding in the stomatal pore. SEM is particularly useful to reveal the rich diversity of horsetail stomata typologies (Figure 4). In some observations, a silicified layer seems to cover a stoma devoid of radiating ribs (or the image may refer to stomata viewed from opposite sides; Figure 4A). In others, it is possible to observe a transversal view cutting a stoma in half (and unveiling the inner structure of overlapping cells; Figure 4B), isolated or pairs of sunken stomata surrounded by silicified papillae, or naked pores apparently distorted probably because they are formed by two adjacent stomata (Figure 4D). 

The extracted silica shows that epidermal cells appear as silicified scales with thick margins (Figure 3E), while parenchymatic cells are larger and have thinner cell walls (Figure 3F). As to whether these features are also observed *in vivo* needs further proof.

**Figure 5:**
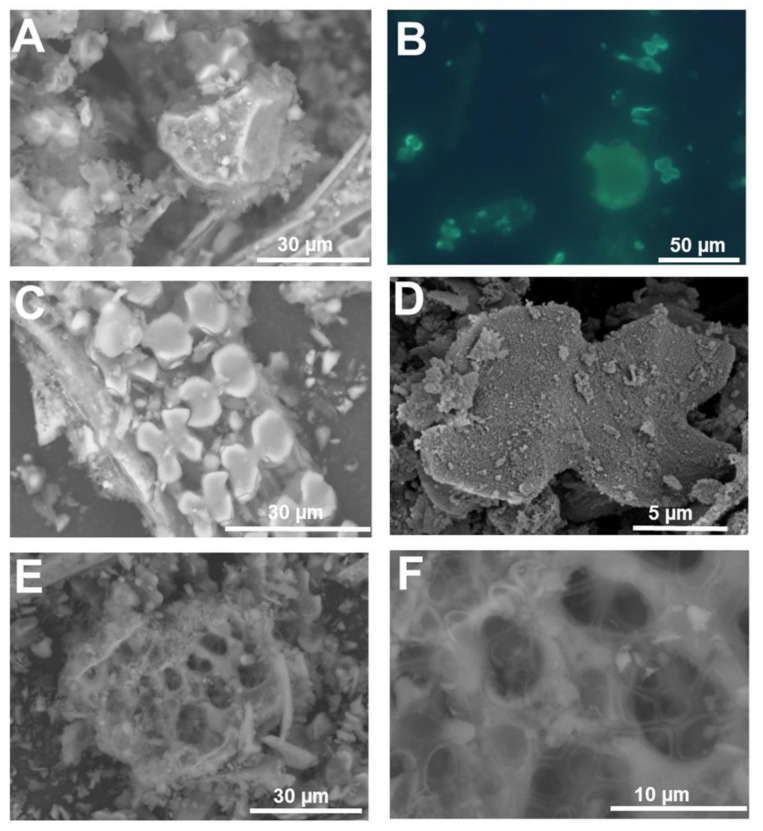
SEM (topographic contrast) and fluorescence microscopy images of the silica extracted from rice leaf. (**A**) Bulliform cells with silicified fibrous material. (**B**) Bulliform cell and silica cells stained with PDMPO. (**C**) Dual-layer of silica cells. (**D**) Fine detail of the silicified granules present on the surface of a silica cell. (**E**,**F**) Probable multicellular silicified pedestal of a rice trichome. Images (**A**,**C**,**E**,**F**) were taken using a HITACHI TM3000 TableTop SEM. Images (**B**) and (**D**) were taken using an Olympus BX50 fluorescence microscope and a Hitachi S-4500 SEM, respectively.

**Figure 6:**
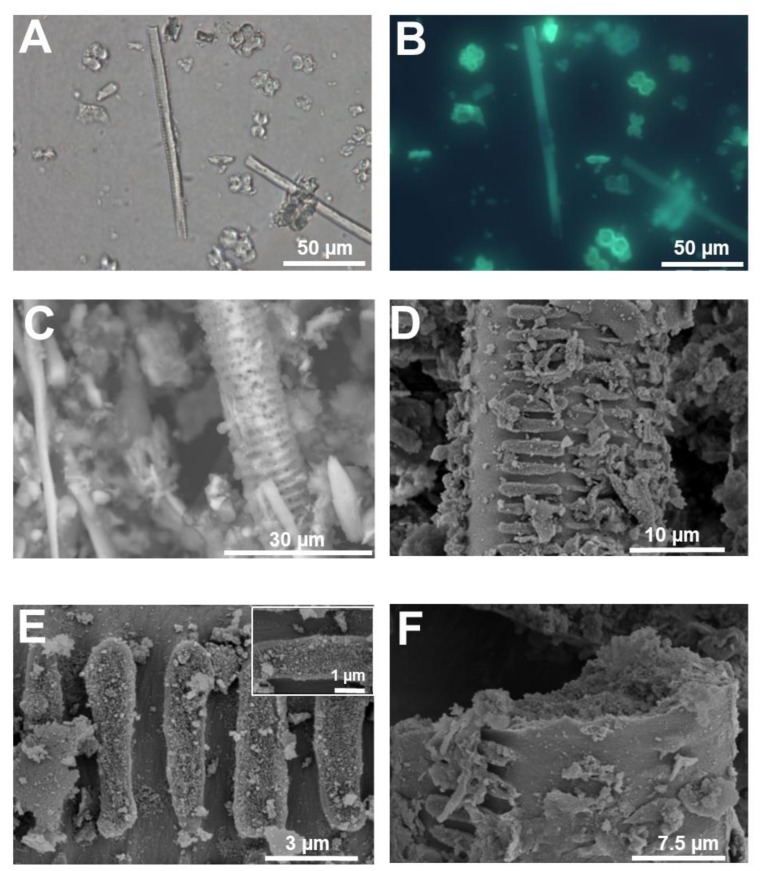
Silicified xylem vessel from rice stem. (**A**) Differential interference contrast and (**B**) fluorescence microscopy after PDMPO staining of silica extracted from rice stem. A xylem vessel is present among silica cells. (**C**) SEM image (topographic contrast) of a rice silicified xylem vessel. (**D**) Details of helical cell wall thickenings. (**E**) Helical thickening of the silicified xylem vessel in (**D**). Inset: fine details of the granular material present on the surface of the thickenings in (**D**). (**F**) Detail of the granular material forming the silicified cell walls of the xylem vessel in (**D**). Images (**D**–**F**) were taken using a Hitachi S-4500 SEM. Images (**A**,**B**) were taken using an Olympus BX50 fluorescence microscope and image (**C**) with a HITACHI TM3000 TableTop SEM.

Microwave acid digestion of rice leaves followed by SEM provided a richness of structural details (Stokes, 2015). Besides known silicified cells, namely bulliform and silica cells (Figure 5A–D), multicellular structures likely corresponding to the silicified pedestal of a trichome were also observed (Figure 5E,F). The extracted silica also revealed less common silicified structures, namely a xylem vessel (Figure 6): in the literature, silicified xylem vessels were reported in one work on oat [48] and barley [49]. Samples observed at the SEM after gold-plating revealed the fine silicified granules covering the ladder-like thickenings of the xylem vessel (Figure 6E,F) and the surface sandy texture of the silica cell (Figure 5D).

The difference in image quality between a benchtop SEM (resolution between 5–30 nm, depending on the model) and a conventional instrument is noteworthy (Figure 3, Figure 4, Figure 5 and Figure 6), but expected given the difference in spatial resolution between the two devices. Benchtop SEM has two main advantages, i.e., both ease of use and ease of sample preparation for insulating samples. Silica is merely mounted on a sticky tab attached to a stub, fixed within the analysis chamber, sealed and then imaged. Since the measurements are carried out in a low vacuum, the build-up of charge on silica surface can be avoided without adding a conductive layer. By comparison, insulating samples, such as silica, must be gold- or carbon-plated before being imaged using a conventional SEM and the procedure for actually using it is more complicated (higher magnification attainable with a resolution up to 1.5 nm) and requires the assistance of a trained staff member.

The two different approaches to SEM imaging can complement each other: samples which have been imaged using a benchtop SEM are compatible with the conventional electron microscope. The easy to handle benchtop instrument can be used to quickly scan over multiple samples, identifying structures of interest which can then later be gold-plated and analysed using the higher resolution SEM. By combining the two instruments, the ease and simplicity of the benchtop SEM can be used to analyse large quantities of digested silica, whilst the higher resolution and better image quality of the conventional SEM can be used to image specific structures.

## 4. Imaging Plant Silica Through Particle Induced X-ray Emission with Focused Beam and Micro-X-Ray Fluorescence Spectrometry

The imaging of elements using X-ray-based techniques improves lateral resolution (down to the sub-micron under vacuum) and sensitivity [50]. Micro-PIXE is a variant of the standard particle-induced X-ray emission (PIXE) technique which accelerates protons or helium ions towards the sample and detects X-rays of characteristic energy unique to each element that are emitted when the sample is hit by the micro-focused ion beam (microprobe) [51]. The technique is non-destructive and versatile, since it allows to image also live specimens by using an external beam [52,53]. However, the use of external or in-air beam is not suitable to detect Si, since the low energy fluorescence signal (1.74 keV) from plant tissues is completely absorbed in air. For this reason, imaging of Si with PIXE requires vacuum conditions, making sample preparation challenging. Under vacuum conditions, only dehydrated plant samples can be measured, or the microprobe should be equipped with cryo-stage enabling measurements on frozen-hydrated tissues. Since only a few microprobes are equipped with cryo-stage [54], the most appropriate way to preserve the distribution, speciation and concentration of elements in a state as close as possible to the native one is via the use of cryotechniques (e.g., cryofixation, followed by cryosectioning and freeze-drying; [55]). 

Micro-PIXE has been used extensively to map element distribution in plant tissues, especially hyperaccumulators that can accumulate high concentrations of metal(loid)s [52]. For example, in *Thlaspi praecox*, a known Cd and Zn accumulator, a study analysed the distribution of Cd, Zn and Pb in leaves [56,57] and seeds and revealed a preferential storage of Zn and Pb in metabolically less active parts of the leaves (epidermis), while Cd is localised in leaf mesophyll. In the embryonic axis Cd and Zn were localised in cotyledons [58].

PIXE was previously used to detect Si in plant tissues challenged by pathogens: the metalloid accumulated at sites of necrotic lesions in *Lagenaria sphaerica* (a member of *Cucurbitaceae*), where it probably has a role in containing the hypersensitive lesions [59].

**Figure 7:**
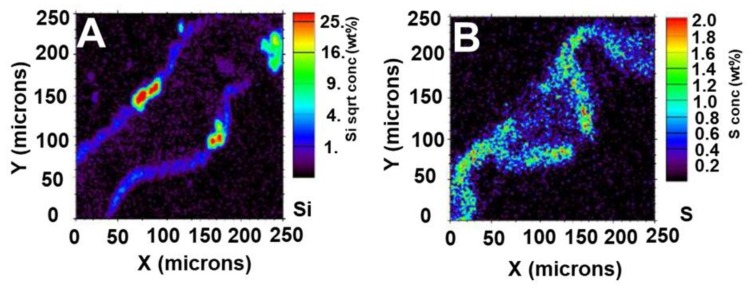
Micro-PIXE images in rice leaf tissue grown with 1 mM silicic acid. (**A**) Silicon detection coinciding with silica cells above and below the vascular bundle. (**B**) Sulphur map. The colour scale on the right side of each image shows the percentage weight detection of each element in that region of the sample. Micro-PIXE analysis was performed at the Jožef Stefan Institute in Slovenia.

Micro-PIXE can be successfully used to detect Si distribution in the leaves of accumulators such as rice, after cryofixation, cryosectioning and freeze-drying [60]. A faint but consistent Si signal can be detected at both the upper and lower epidermis, consistently reading at 3% weight (Figure 7A). Strong Si signals are clear both above and below the vascular bundle peaking at 25% weight, indicative of silica cells (Figure 7A). Si accumulation on the abaxial and adaxial sides was already proven in rice using SEM coupled to energy dispersive X-ray analysis (EDX), which allows a comparison of Si abundance among samples receiving different treatments [61]. Recently, PDMPO staining of microwave acid digested samples also showed accumulation of Si on the epidermis of rice leaves [20].

XRF has a higher resolution and better sensitivity as compared to PIXE and also allows analysis *in vivo* [62], which is, in case of Si, once again challenging due to the low energy of Si Kα X-ray fluorescence line.

Si distribution was studied via XRF in the grasses *Phragmites australis*, *Phalaris arundinacea*, *Molinia caerulea*, *Deschampsia cespitosa* and in the sedge *Carex elata*, in combination with the analysis of the leaf optical properties [63]. Grasses were shown to contain Si for >1% of the dry mass, while sedges contained 0.4%. Structures like prickle-hairs and epidermal tissues that were highly encrusted with Si significantly affected light transmittance. Both benchtop and synchrotron-based XRF microscopy exist; however, the latter, despite the limitation due to the access to synchrotron facilities, is faster, because of much brighter excitation beams, an important parameter when imaging hydrated plant tissues [62]. It should however be noted that the drawback of using such brighter beams is the damage to the samples due to the radiation, which is particularly prominent in the low energy range. These damages, while not affecting substantially the morphology of the samples, affect the biochemical profile by causing biomolecules’ ionization [64]. The possibility of using low-energy XRF provides higher sensitivity for elements with lower atomic number like C, O, N, Si and Al: a study on tea plants used low-energy X-ray fluorescence (LEXRF) to image Al and low atomic number elements within leaves [65]. Evidence for the colocalization of Si and Al was also observed in *Sorghum* root tissues using LEXRF, thereby indicating a role for Si in Al detoxification [66].

**Figure 8:**
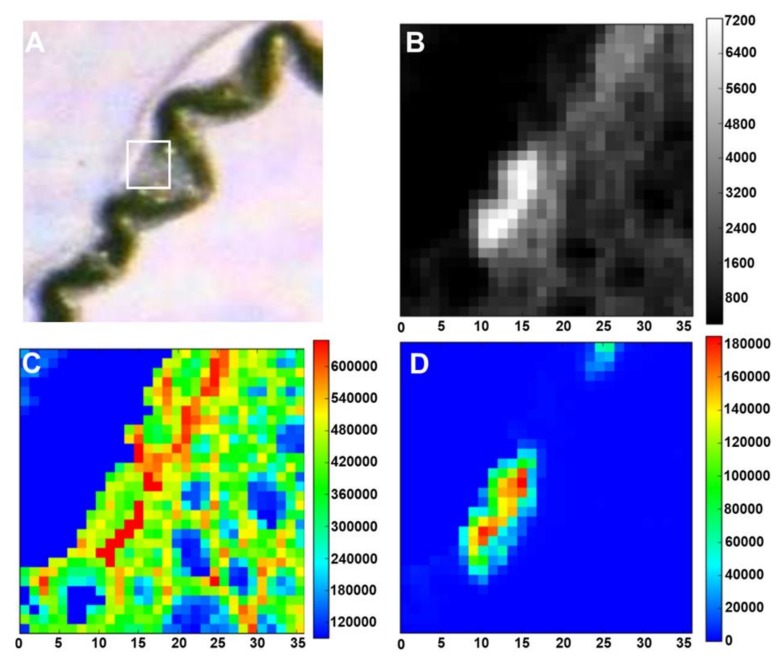
Light microscope and low-energy X-ray fluorescence (LEXRF) imaging of rice leaf tissue grown with 1 mM silicic acid. (**A**) Light microscope image showing the rice leaf sample; the white box highlights LEXRF imaging area. (**B**) Areal density image (µg·cm^−2^) of sample. (**C**) LEXRF detection of oxygen (µg·g^−1^) providing an overview of the plant tissue. (**D**) LEXRF detection of silicon (µg·g^−1^) above the vascular bundle and coinciding with silica cells. LEXRF images show the amount of the relevant element (oxygen or silicon) in μg·cm^−2^. The colour scale on the right side of each image is a scale of intensity of each element in that region of the sample. The LEXRF measurements were performed using the TwinMic X-ray fluorescence spectro-microscope at the Elettra Synchrotron Radiation Facility in Trieste. Quantification was performed according to Kump and Vogel-Mikuš [67]. X-axis is in µm.

By using LEXRF, we successfully detected Si within the leaves of rice [60]. A strong signal peaking at around 180 mg·g^−1^ was found above the vascular bundle. The location and dumbbell appearance suggest that the element observed is a silica cell (Figure 8D). 

## 5. Mass Spectrometry Imaging of Si: SIMS Nano-Analysis

SIMS nano-analysis is a chemical imaging technique characterised by a high spatial resolution (down to 50 nm; [34]) and a high sensitivity (below one part per million; for more details, please refer to Sangely et al. [35]). It is based on the detection of secondary ions generated after having bombarded and eroded the specimen’s surface with a high energy primary ion-beam (16keV, Cs^+^ or O^−^). The secondary ions are analysed by a mass spectrometer. The measurement is carried out under ultra-high vacuum conditions. During sputtering, the acquisition of successive images also allows to obtain a subsequent reconstruction of the volume sputtered (3D imaging).

SIMS already proved to be a good complementary imaging technique for determining the distribution of elements in plant tissue [34,68]. Sample preparation is a crucial step to achieve a representative distribution of elements in samples and involves cryofixation. The challenge lies in the fact that it is necessary to remove water from the sample, while keeping an intact structure and preventing the redistribution of mobile elements [69].

SIMS was used on rice to map Si distribution [70] in wild-type and *lsi2* mutants: the metalloid was found to accumulate at higher levels in the proximal side of the root endodermis, in a region of the cell wall coinciding with the presence of a dark layer as imaged by transmission electron microscope (TEM). SIMS was also used to map the distribution of Si and germanium (Ge) in the roots and leaves of the grasses *Poa annua* and *Dactylis glomerata* [71]: the signal overlapped in the endodermal cells, close to the suberized Casparian strip, while in the leaves germanium was barely present.

SIMS nano-analysis was recently used to map Si in the accumulator horsetail [19]. The presence of a Si double layer was detected on the outer leaf side and in regions close to stem furrows, while a single continuous layer was present on the inner epidermis of the leaf side facing the stem node [19]. SIMS nano-analysis revealed that the regions showing the double Si layer contained organic material in-between. Interestingly, a single layer of Si was detected in a probable non-epidermal internal cell layer.

**Figure 9:**
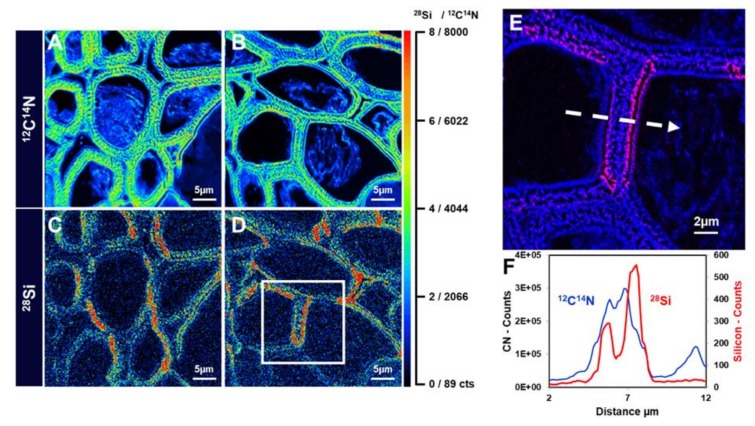
NanoSIMS images of hemp hypocotyl. (**A**) and (**B**) Distribution of ^12^C^14^N^−^ in the bast fibres of textile hemp grown for 15 days on 1mM sodium metasilicate. (**C**) and (**D**) ^28^Si^−^ in the bast fibres of textile hemp grown for 15 days on 1mM sodium metasilicate. (**E**) Zoomed image of the white box in (**D**). (**F**) Linescans of the ^12^C^14^N and ^28^Si in (**E**) (relative to the region marked with the white dotted arrow). The colour scale goes from black to red with increasing intensity. The scale bars are 5 µm in panels (**A**–**D**) and 2 µm in panel (**E**). Images were taken using a CAMECA NanoSIMS 50 instrument (Gennevilliers, France).

High-resolution SIMS imaging was also used recently to map Si distribution in the stem tissues of textile hemp [9]. A signal was observed in the cell walls of xylem vessels in samples prepared from plantlets grown in the presence of sodium metasilicate 1 mM and in the cell walls of bast fibers too, preferentially on the side facing the stem cortex (Figure 9). The accumulation of Si seems to be associated within a region of the gelatinous layer closest to the fibre lumen. The linescan of the ^12^C^14^N signal indicates the gelatinous organic layer which overlaps with the ^28^Si linescan (Figure 9F). This suggests a mechanical role of Si and opens up the way to future investigations aimed at understanding where exactly in the gelatinous layer silica is deposited.

## 6. Future Perspectives

Our understanding of plant silicification is still partial and many open questions remain, especially in relation to which macromolecules can template silica deposition. The majority of the studies available in the recent literature have focused on the identification and functional characterisation of the membrane channels mediating the entry of silicic acid. Only a handful of studies, some of which date back to the early 80s, have analysed the spectacular silicified structures of plants. Molecular approaches can be used together with fluorescence microscopy after PDMPO staining, elemental imaging or electron microscopy following microwave acid digestion to infer the role of candidate genes in plant silicification: databases providing a collection of mutants of known silicifiers, such as rice [72] or *Sorghum* [73], will be useful resources. For example, plant cell mutants can provide important insights into the relationship between specific cell wall macromolecules and silica accumulation.

## Figures and Tables

**Table 1:** Summary of the techniques surveyed in the present review for the study of plant silicification. Details of the information provided, advantages/drawbacks and spatial resolution are schematically given.

Technique	Information	Advantages	Drawbacks	Spatial Resolution
**Optical microscopy (dyes)**	Tissue distribution	Instrument accessible in any laboratory	Invasive (tissue fixation) and slicing; etching; lack of discrimination between lignin and silica	200 nm lateral 600 nm axial [31]
**Confocal microscopy**	Tissues distribution	Thick samples	Background fluorescence of chloroplasts	180 nm lateral 500 nm axial [32]
**Tabletop scanning electron microscopy (SEM) (on extracted silica)**	Secondary electron images (SE): structure	Easy to use, rapidity;no sample preparation	Lower resolution than conventional SEM	5–30 nm (depending on the model)
**Conventional SEM (on extracted silica)**	SE images: very thin structure	High resolution	Lengthy procedure for sample preparation with the use of hazardous chemicals	1 nm
**Micro-PIXE (microbeam particle-induced X-ray emission)**	Quantitative elemental mapping	Quantification	Limit in the analysis of some light element (F, Li, B); time devoted to cryotechniques to preserve a state as close as possible to the native one	1 µm
**LEXRF (low-energy X-ray fluorescence)**	Quantitative elemental mapping	Quantification	Limited access to synchrotron facilities; time devoted to cryotechniques for analysis of plant tissues	100 nm [33]
**NanoSIMS**	Elemental mapping	High sensitivityHigh lateral resolution3D imaging	Sample preparation (removal of water without causing diffusion of element and damage of the sample structure)	50 nm [34,35]
